# Advancing Antimicrobial Resistance Research Through Quantitative Modeling and Synthetic Biology

**DOI:** 10.3389/fbioe.2020.583415

**Published:** 2020-09-18

**Authors:** Kevin S. Farquhar, Harold Flohr, Daniel A. Charlebois

**Affiliations:** ^1^Precision for Medicine, Houston, TX, United States; ^2^Department of Physics, University of Alberta, Edmonton, AB, Canada

**Keywords:** antimicrobial resistance, gene regulatory networks, mathematical modeling and simulation, non-genetic heterogeneity, stochastic gene expression, synthetic biology

## Abstract

Antimicrobial resistance (AMR) is an emerging global health crisis that is undermining advances in modern medicine and, if unmitigated, threatens to kill 10 million people per year worldwide by 2050. Research over the last decade has demonstrated that the differences between genetically identical cells in the same environment can lead to drug resistance. Fluctuations in gene expression, modulated by gene regulatory networks, can lead to non-genetic heterogeneity that results in the fractional killing of microbial populations causing drug therapies to fail; this non-genetic drug resistance can enhance the probability of acquiring genetic drug resistance mutations. Mathematical models of gene networks can elucidate general principles underlying drug resistance, predict the evolution of resistance, and guide drug resistance experiments in the laboratory. Cells genetically engineered to carry synthetic gene networks regulating drug resistance genes allow for controlled, quantitative experiments on the role of non-genetic heterogeneity in the development of drug resistance. In this perspective article, we emphasize the contributions that mathematical, computational, and synthetic gene network models play in advancing our understanding of AMR to discover effective therapies against drug-resistant infections.

## Introduction

Antimicrobial resistance (AMR) is an emerging health crisis that is undermining modern medicine (World Health Organization, 2014). AMR arises when bacteria, fungi, viruses or other microbes no longer respond to the antimicrobial drugs used to treat them. As of 2016, 700,000 deaths per year are attributed to AMR (O’Neill and The Review on Antimicrobial Resistance, 2016). If unmitigated, it is estimated that by 2050, AMR will kill 10 million people per year globally and result in a cumulative lost global production cost of 100 trillion USD. Though it has been argued that these figures may be over-estimates (de Kraker et al., 2016), there is undoubtedly a large and increasing clinical and public health burden associated with AMR. Drug resistance during chemotherapy also continues to be the major limiting factor for successfully treating patients with cancer (Vasan et al., 2019). In order to mitigate drug resistance, we need to establish new quantitative tools to study the drug resistance process, to discover new drugs, and to develop novel treatment strategies that extend the “lifespan” of antimicrobial and chemotherapy drugs.

**FIGURE 1:**
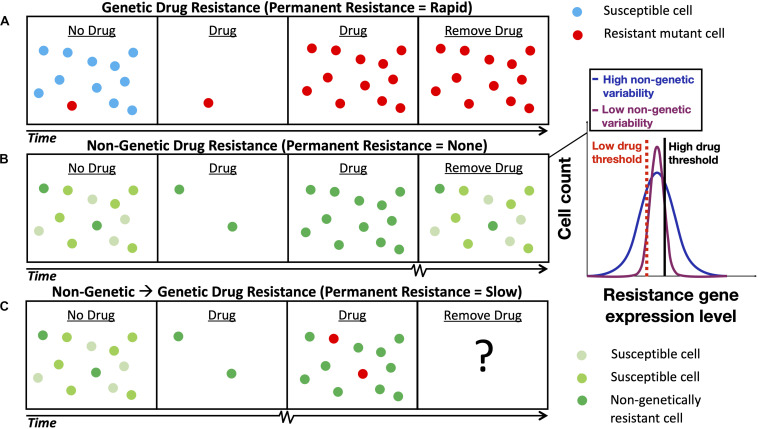
Schematic depicting the development of non-genetic and genetic drug resistance. **(A)** The development of genetic drug resistance via evolution by natural selection of a pre-existing drug resistance mutation. **(B)** The development of non-genetic drug resistance in a clonal cell population via the selection cells with sufficiently long gene expression fluctuation timescales. The shade of green denotes the level of gene expression of a drug resistance gene inside the cell; lighter and darker shades of green represent lower levels and higher levels of gene expression, respectively. Inset illustrates gene expression histograms typically obtained from clonal cell populations with low and high levels of non-genetic, cell-to-cell variability. A high level of non-genetic variability is advantageous at high drug concentrations and a low level of non-genetic variability is advantageous at low drug concentrations (cells with resistance gene expression levels below a given drug threshold perish). **(C)** The evolution of longer-term, genetic drug resistance is facilitated by shorter-term, non-genetic drug resistance; the ultimate outcome will be determined based on the condition-dependent relative fitness of each subpopulation. Squiggly lines on the time axes in **(B,C)** represent longer timescales. Notably, the relaxation timescales of non-genetic fluctuations in the expression of drug resistance genes regulated by positive feedback gene circuits have been estimated to be 58 h in mammalian cells (Farquhar et al., 2019) and 283 h in yeast (Nevozhay et al., 2012).

**FIGURE 2:**
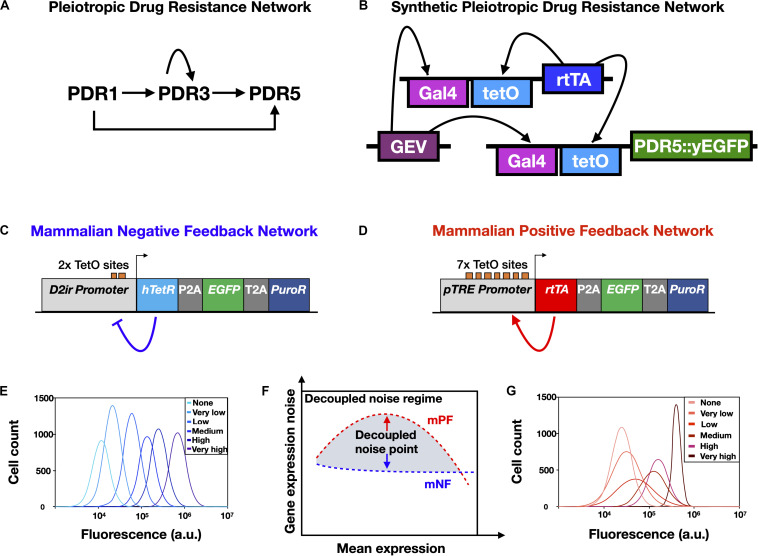
Synthetic gene networks engineered to mimic natural drug resistance gene networks and study the effects of non-genetic heterogeneity on AMR. **(A)** Yeast pleiotropic drug resistance (PDR) gene network. Imbedded in this gene network structure are positive feedback regulation (self-activation of PDR3) and feedforward regulation (PDR1 indirectly activates PDR5 through PDR3) and direct activation (PDR1 activates PDR5). **(B)** A synthetic gene network engineered to have the same network motif as the PDR network shown in **(A)**. Note that fluorescence reporter genes, such as yEGFP, are fused to drug resistance genes, such as PDR5, to enable experimental measurement. **(C)** Schematic of the mammalian negative feedback (mNF) gene network, which expresses the humanized tetracycline repressor (hTetR) gene, the puromycin resistance gene (PuroR), and the fluorescence reporter EGFP separated by self-cleaving 2A elements. **(D)** Schematic of the mammalian positive feedback (mPF) gene network, which expresses the reverse tetracycline transactivator (rtTA), PuroR, and EGFP separated by self-cleaving 2A elements. **(E)** Schematic of representative gene expression histograms obtained from a dose response of the mNF strain. **(F)** The decoupled noise regime (gray shading) is composed of decoupled noise points (red and blue arrows), which occur where the mean gene expression noise for the high-noise mPF and the low-noise mNF gene networks are decoupled from mean gene expression. **(G)** Schematic of representative gene expression histograms obtained from a dose response of the mPF strain. The legends in **(E,G)** indicate the inducer (doxycycline) level for each distribution. Panel **(A)** was reproduced from Charlebois et al. (2014) with permission, panel **(B)** was adapted with permission from Camellato (2018), and panels **(C–G)** were adapted from Farquhar et al. (2019), CC BY 4.0 (https://creativecommons.org/licenses/by/4.0/).

It is well established that drug resistance can develop through genetic mutation (Figure 1A) that causes a permanent change in a micro-organism’s DNA or through the acquisition of a drug resistance gene (e.g., horizontal gene transfer that occurs in bacteria) (Ochman et al., 2000). More recently, research has uncovered a new form of non-genetic stress resistance that can arise from fluctuations in gene expression in clonal cell populations (Figure 1B; Fraser and Kærn, 2009; Geiler-Samerotte et al., 2013; van Boxtel et al., 2017); this, for example, includes the non-genetic drug resistance associated with the increased expression of genes that encode efflux proteins that pump antimicrobial drugs out of pathogenic yeasts such as *Candida glabrata* (Ben-Ami et al., 2016) and *Cryptococcus neoformans* (Mondon et al., 1999). Targeting this phenomenon will be important for mitigating AMR, as some non-genetically drug-resistant pathogens are not easily detected by standard laboratory tests (Sears and Schwartz, 2017) and non-genetic drug resistance may be associated with the failure of antimicrobial therapies (Ben-Ami et al., 2016; Wuyts et al., 2018) and chemotherapies (Brock et al., 2009). Non-genetic heterogeneity resulting in drug resistance has been shown to be modulated by gene regulatory network structure (e.g., in the PDR network discussed below) (Charlebois et al., 2014; Inde and Dixon, 2018; Camellato et al., 2019) and may accumulate through multiple slightly asymmetric cell divisions (Mitchell et al., 2018; Tripathi et al., 2020). The emerging paradigm is that drug resistance is a multi-stage process and that acute, non-genetic drug resistance can facilitate the evolution of permanent, genetic drug resistance (Figure 1C). Non-genetic mechanisms are now thought to facilitate genetic resistance by increasing the population size under drug treatment and hence the chance of acquiring genetic mutations (Brock et al., 2009; Charlebois et al., 2011; Farquhar et al., 2019), and through synergism between adaptive mutations and non-genetic heterogeneity (Bódi et al., 2017; Salgia and Kulkarni, 2018). Furthermore, it is known that mutations in PDR1, a gene that regulates PDR5 in the pleiotropic drug resistance (PDR) network in yeast (Figure 2A), can cause full resistance to the antifungal drug fluconazole (Ferrari et al., 2009). Though, more research is needed to elucidate the interplay between non-genetic and genetic forms of drug resistance.

Mathematical models of drug resistance have been used for over three decades (Lavi et al., 2012); many older mathematical studies were based on ABC (ATP-binding cassette) transporters, such as the PDR5 gene that is regulated by PDR1 in the PDR network, as the main mechanism of resistance. These models are now beginning to include more contemporary knowledge of AMR mechanisms and incorporate how drug resistance gene networks function and evolve during treatment (Charlebois et al., 2014; Farquhar et al., 2019). Mathematical models have the potential to predict the effectiveness of various treatment strategies, such as using combination drug therapies to overcome AMR (Baym et al., 2016), and can guide laboratory experiments by identifying experimental targets and by narrowing down the immense number of ways that antimicrobial drugs can be applied. Additionally, these models can elucidate mechanisms underlying the development of AMR (e.g., Farquhar et al., 2019) and predict AMR from experimental data (Arepyeva et al., 2017).

Synthetic biology is rapidly becoming part of the solution to many of our needs in medicine, agriculture, and energy production (El Karoui et al., 2019). A particularly promising application is to genetically engineer micro-organisms to carry synthetic gene networks to study AMR in a more quantitative, controlled, and efficient manner than has been possible using traditional (“natural” or non-genetically modified) model micro-organisms (González et al., 2015). At present, it is extremely challenging to develop and experimentally validate mathematical models using pathogens, where drug resistance genes have evolved to be highly connected to the host genome; for instance, the expression of MDR1/p-glycoprotein (responsible for multiple drug resistance (MDR) of tumors to chemotherapy; Gottesman et al., 2002) is regulated by a multitude of factors, making it difficult to quantitatively study how non-genetic mechanisms may contribute to AMR and drug resistance in cancer (Camellato et al., 2019). Furthermore, unlike synthetic drug resistance networks, many native resistance networks are still not known completely. Nevertheless, the design of synthetic gene networks is a model-guided process (Sakurai and Hori, 2018) and these networks are constructed to mimic known natural drug resistance networks using techniques from genetic engineering (Cameron et al., 2014; Bartley et al., 2017).

## Mathematical Modeling of Non-Genetic Antimicrobial Resistance

### Modeling Non-genetic Gene Expression Heterogeneity in Drug Resistance

Early work on non-genetic drug resistance focused on the amplitude of fluctuations or “noise” in the expression of drug resistance genes. Models predicted that low gene expression noise would be beneficial under low drug concentrations and that high gene expression noise would be beneficial under high drug concentrations (Figure 1B, inset; Blake et al., 2006; Zhuravel et al., 2010); these predictions were confirmed experimentally in the same studies. Subsequently, a more general theoretical framework was developed that incorporated the frequency of gene expression noise, as well as the amplitude of the expression noise (Charlebois et al., 2011). Importantly, using this quantitative framework it was hypothesized that drug resistance can develop independently of mutation, provided that the fluctuation timescales are sufficiently long. Cell population models (Arino and Kimmel, 1993; Henson, 2003; Charlebois and Balázsi, 2019) have also been used to incorporate the multi-scale nature of AMR. For instance, a stochastic model of gene expression was combined with a population simulation algorithm to computationally investigate the evolution of gene expression noise (Charlebois, 2015).

### Modeling Drug Resistance Networks in Microbes and Mammalian Cells

Mathematical models have been used to investigate the effect gene network structures or motifs have on AMR. For instance, it was shown computationally that gene network motifs can enhance drug resistance by modulating gene expression noise within a cell population (Charlebois et al., 2014). Charlebois et al. showed that feedforward and positive feedback loops, the same network motifs that have been found to be imbedded in some gene networks regulating AMR in pathogenic yeast (Kolaczkowski et al., 1998) and human cancer cells (Misra et al., 2005), enhance drug resistance *in silico*. This new understanding of how gene network structure regulates AMR opens up new lines of research and identifies new potential therapeutic targets (e.g., targeting regulator genes in the network, rather than the drug resistance genes they control) against drug-resistant pathogens and cancers to be investigated experimentally.

Mathematical modeling and computer simulations have been used to predict how drug efflux pump proteins affect gene network function and fitness in prokaryotic and eukaryotic organisms. In Langevin and Dunlop (2018) it was found experimentally that the cellular fitness benefit of AcrAB-TolC, a well-known multi-drug resistance pump in *E. coli*, depended on the rate of stress induction; fits to data allowed the fitness benefit that the pumps conferred under different stress induction rates to be accurately predicted by mathematical models. In another study, it was predicted that incorporating negative feedback via drug efflux pumps into synthetic gene networks can increase the response of the gene network at low antibiotic inducer concentrations (Diao et al., 2016). This prediction was confirmed experimentally in the same study using synthetic gene networks in *S. cerevisiae* and was found to be the result of reduced regulator gene expression.

In Farquhar et al. (2019) the authors developed a stochastic population dynamics model to infer mechanisms for drug resistance in mammalian cells. The stochastic population model predicted that gene network motifs facilitate the development of acute drug resistance and that non- or slow-growing subpopulations of “persister-like” cells that do not succumb are critical reservoirs for the development of fast growing, heritably drug-resistant mutants enabling longer-term drug resistance (see Brauner et al., 2016; Rosenberg et al., 2018; Berman and Krysan, 2020 for the distinction between “tolerance,” “heteroresistance” or “persistence,” and “resistance”). This study compliments previous work in bacteria that demonstrated that antibiotic tolerant non- or slow-growing mutant cells precede the developed genetic drug resistance during intermittent antibiotic exposure (Levin-Reisman et al., 2017). The persistence phenotype (e.g., Kussell et al., 2005) and stochastic phenotype switching (e.g., Acar et al., 2008) have also been investigated in mathematical models and experiments on genetically engineered micro-organisms and found to affect fitness in fluctuating environments.

Ultimately, mathematical and computation models of AMR must be validated by performing quantitative drug resistance experiments; genetically engineered cells that harbor synthetic gene networks controlling the expression of drug resistance genes is proving to be an effective experimental model system.

## Synthetic Drug Resistance Gene Networks and Antimicrobial Resistance Experiments

Genetic engineering techniques are used to synthetize and combine DNA to build synthetic gene networks or “circuits” (Cameron et al., 2014) that control drug resistance genes. Common methods used to engineer synthetic gene networks include recombinant molecular cloning, Gibson assembly (Gibson et al., 2009; Santos-Moreno and Schaerli, 2019), and CRISPR-Cas9 gene editing (Nissim et al., 2014; Jusiak et al., 2016). Cell-to-cell heterogeneity may cause unexpected deviations from intended synthetic gene circuit behavior (Beach et al., 2017). However, statistical tools can enhance the design process and reliability of synthetic gene networks (Sakurai and Hori, 2018). With proper design, synthetic gene networks can be precisely tuned to control gene expression mean and noise levels using chemical inducers that do not adversely affect the micro-organisms harboring these networks.

### Synthetic Antimicrobial Resistance Gene Networks

Synthetic gene networks have been engineered to regulate drug resistance and have been shown to serve as well-characterized models of natural stress response modules in evolution experiments (González et al., 2015; Bódi et al., 2017; Farquhar et al., 2019; Gouda et al., 2019).

Nevozhay et al. (2012) constructed a two-gene positive feedback network that enables bi-stable gene expression to control a Zeocin antibiotic resistance gene in *S. cerevisiae*.

In this work, a computational approach based on stochastic cellular movement in “gene expression space” was used to predict cell population fitness of low- and high-expressing subpopulations. The authors found an optimum on the fitness landscape that balances the costs and benefits of expressing a drug resistance gene in various experimental antibiotic inducer and drug conditions. In a subsequent microbial evolution study using the same positive feedback yeast strain, it was found that the synthetic gene network was fine-tuned by evolution to modulate the network’s noisy response and optimize fitness via specific “intra-circuit” and “extra-circuit” DNA mutations (González et al., 2015), which can lead to loss of gene circuit function that can be regained in certain conditions under drug selection (Gouda et al., 2019). The study by Gouda et al. (2019) also suggests that slow growth due to antibiotics may allow cells to access hidden drug-resistant states prior to genetic changes. Computational models based on fitness and gene expression properties have been developed to predict specific aspects of evolutionary dynamics (including the speed at which the ancestral genome disappears from the population and the types and number of mutant alleles that establish in each experimental condition) in different inducer and drug conditions (González et al., 2015). These computational models were validated in the same studies by microbial evolution experiments on the genetically engineered positive feedback yeast strain (Nevozhay et al., 2012; González et al., 2015; Gouda et al., 2019).

Genetically engineered networks have also been designed to control the expression of genes that encode efflux proteins that lead to AMR. Diao et al. (2016) used synthetic negative feedback gene networks, inducible by the antibiotic doxycycline, to regulate the expression of PDR5. This study found that the addition of a second layer of negative feedback (resulting from pumping doxycycline out of the cell by the PDR5 protein) altered the dose-responses of the original gene circuits in a manner that was predictable by mathematical modeling. In another study, Camellato et al. (2019) engineered a synthetic gene network in yeast to mimic the PDR5 and MDR1 networks that underly multi-drug resistance in yeast and human breast cancer cells (Figure 2B). In agreement with computational predictions made years earlier (Charlebois et al., 2014), the authors found that feedforward and positive feedback network motifs enabled rapid, self-sustained activation of gene expression leading to enhanced cell survival in the presence of a cytotoxic drug. It has been proposed that activating the expression of genes that encode multi-drug resistance efflux pump proteins in the absence of antibiotic pressure may allow susceptible bacteria to outcompete resistant bacteria, which normally down-regulate the expression of resistance genes in conditions without antibiotics to eliminate the associated fitness cost (Wang et al., 2019).

### Synthetic Drug Resistance Gene Circuits in Mammalian Cells

To experimentally investigate the role of non-genetic cell-to-cell variability in cancer drug resistance, it is imperative to precisely control this non-genetic heterogeneity that can drive adaptation to chemotherapeutic agents. Synthetic gene circuits integrated in mammalian cells can be designed to precisely control gene expression noise for drug resistance genes, while keeping the mean expression levels the same (Figure 2F; Aranda-Díaz et al., 2017; Farquhar et al., 2019). This approach allows synthetic gene circuits to separate key biological variables contributing to resistance from other confounding variables like mean expression and genetic background.

In Chinese Hamster Ovary (CHO) cells with a recombinase-mediated integration site known as a “Flp-In” landing pad (Wirth and Hauser, 2004), Farquhar et al. (2019) designed, constructed, and integrated into the landing pad a mammalian negative feedback (mNF) synthetic gene circuit (Figure 2C) based on a humanized tetracycline repressor (hTetR) gene (Nevozhay et al., 2013); the mNF circuit demonstrated doxycycline-inducible expression of a purmoycin drug resistance gene (PuroR) with low gene expression noise (Figure 2E). Highlighting the advantages of mathematical modeling in synthetic gene circuit design, the mNF circuit was based on another gene circuit transferred that applied modeling to predict the effects of multiple design iterations, leading to increased fold change and minimal gene expression noise (Nevozhay et al., 2013). Complementing the low noise mNF gene circuit, Farquhar et al. (2019) also constructed a mammalian positive feedback (mPF) gene circuit (Figure 2D) regulated by a reverse tetracycline trans-activator (rtTA), integrated into the same CHO genomic integration site as the low-noise mNF circuit, leading to doxycycline-inducible expression of PuroR with high levels of gene expression noise (Figure 2G). Notably, no bimodal gene expression regime was observed for the mPF gene circuit at intermediate doxycycline concentrations, though bimodality is observed for the PF gene circuit in yeast (e.g., Nevozhay et al., 2012). Though the exact mechanism for the lack of observed bimodality in the mPF gene circuit remains unknown, this highlights that genetically engineered circuits do not always function the same way in different organisms. When inducing the two circuits in mammalian CHO cells to express the same PuroR mean expression level (Figure 2F) and treating the CHO cells with various concentrations of puromycin, the authors found that adaptation to low concentrations of drug was more rapid for the mNF circuit with low gene expression noise. On the other hand, high gene expression noise from the mPF circuit facilitated adaptation to high levels of puromycin, while cells with the mNF circuit treated at a high puromycin concentration did not adapt at all. This validated the approach to investigating drug resistance and noise in mammalian cells using synthetic gene networks, which allowed gene expression noise to be decoupled from mean drug resistance gene expression in isogenic cells; this approach could also help to further elucidate the role of rare-cell expression and drug-induced reprogramming in mammalian drug resistance (Shaffer et al., 2017).

DNA sequencing of the gene circuits after adaptation to puromycin and monitoring expression and survival after temporary removal of drug revealed adaptation mechanisms (Farquhar et al., 2019). The self-repression from the tetracycline repressor in the mNF circuit tended to break down through intra-circuit mutations, leading to higher PuroR expression and irreversible resistance to puromycin even without circuit induction. In the mPF circuit, no intra-circuit mutations were found despite PuroR expression levels remaining elevated above pre-treatment mean expression levels, which was reversible and led to re-sensitization to puromycin after inducer removal. Epigenetic factors and chromatin modifications may have driven the elevated expression at the genomic locus which was evolutionarily selected for during adaptation (Berger, 2007). By using synthetic gene networks containing a drug resistance gene in isogenic mammalian cells, Farquhar et al. addressed a long-standing question regarding how non-genetic heterogeneity could lead to initial cell survival during chemotherapy which then facilitates the development of genetic drug resistance in cancer (Brock et al., 2009).

## Discussion

A new interdisciplinary field of research is emerging that combines multi-scale quantitative models with synthetic biology to rationally design gene networks using engineering principles for AMR research. One important goal is to use these models to predict the effects of non-genetic drug resistance on the evolution of genetic drug resistance. Another important goal is to advance pharmaceutical and clinical AMR research by investigating new “resistance proof” antimicrobial compounds and novel therapeutic treatment strategies.

Moving forward, a challenge that must be addressed is how to adapt the mathematical models and translate the experimental discoveries made using synthetic systems to pathogens with complex and highly interconnected gene regulatory networks. More research on pathogenic micro-organisms and mammalian cells is needed to elucidate the underpinnings of non-genetic resistance at the molecular and single-cell levels. Specifically, capturing the complexity of native resistance mechanisms, which are not completely understood, with synthetic gene networks presents both an obstacle and an opportunity. Quantifying known drug resistance effects in genetically engineered organisms may elucidate native resistance mechanisms in pathogens. One possible approach involves adding additional regulatory interactions iteratively to a well-understood synthetic gene network controlling a drug resistance gene and making predictions for their impact on drug resistance. Eventually, the networks become complex enough to mimic the phenotypes caused by native resistance mechanisms. In the cases of cancer subtypes, introducing synthetic gene circuits controlling a gene with specific mutations associated with chemotherapy resistance will be challenging, with genomic instability possibly corrupting the gene circuit. Targeted genomic integration in various cancer cell lines and primary cell strains will also differ in efficiency, making comparisons between cell types difficult. To address these challenges, a better understanding of the gene regulatory networks, mutations, and signal transduction pathways associated with chemoresistance in specific cancers is needed.

Research incorporating quantitative modeling and genetically engineered networks will be critical to fully understand how non-genetic and genetic mechanisms interact in the development of drug resistance, and to discover effective strategies that target acute non-genetic drug resistance to alleviate the development of permanent genetic drug resistance in infectious diseases and cancers. Several promising approaches include synergistically using noise-enhancing compounds to reactivate latent HIV to increase sensitivity to existing antiviral drugs (Dar et al., 2014), using combined drug treatment regimens to target non-proliferating *M. tuberculosis* persisters to reduce treatment times (Zhang et al., 2012), eliminating bacterial persisters using engineering approaches that target bacterial metabolism (Allison et al., 2011), and the use of epigenetic modifiers in combination with targeted therapies to reduce the ability of a cancerous cell to switch phenotypes to acquire a drug-resistant state (Salgia and Kulkarni, 2018).

Overall, combining mathematical models and synthetic gene networks is leading to new quantitative model systems for drug resistance research, which are desperately needed to advance our fundamental understanding of the multi-stage drug resistance process. Ultimately it remains to be seen how discoveries made using these quantitative model systems will translate to pathogens and cancer. However, the potential of this new area of research to help mitigate the socio-economic costs of drug resistance warrants its relentless pursuit.

## Author Contributions

DC conceptualized and supervised the study. KF, HF, and DC contributed to the literature review. HF and DC developed the figures. KF and DC wrote the manuscript. All authors contributed to the article and approved the submitted version.

## Conflict of Interest

KF was employed by the company Precision for Medicine. The remaining authors declare that the research was conducted in the absence of any commercial or financial relationships that could be construed as a potential conflict of interest.

## References

[B1] Acar M. Mettetal J. T. van Oudenaarden A. (2008). Stochastic switching as a survival strategy in fluctuating environments. *Nat. Genet.* 40 471–475. 10.1038/ng.110 18362885

[B2] Allison K. R. Brynildsen M. P. Collins J. J. (2011). Metabolite-enabled eradication of bacterial persisters by aminoglycosides. *Nature* 473 216–221. 10.1038/nature1006 21562562 PMC3145328

[B3] Aranda-Díaz A. Mace K. Zuleta I. Harrigan P. El-Samad H. (2017). Robust Synthetic Circuits for Two-Dimensional Control of Gene Expression in Yeast. *ACS Synth. Biol.* 6 545–554. 10.1021/acssynbio.6b00251 27930885 PMC5507677

[B4] Arepyeva M. A. Kolbin A. S. Sidorenko S. V. Lawson R. Kurylev A. A. Balykina Y. E. (2017). A mathematical model for predicting the development of bacterial resistance based on the relationship between the level of antimicrobial resistance and the volume of antibiotic consumption. *J. Glob. Antimicrob Resist.* 8 148–156. 10.1016/j.jgar.2016.11.010 28167308

[B5] Arino O. Kimmel M. (1993). Comparison of approaches to modeling of cell population dynamics. *SIAM J. Appl. Math.* 53 1480–1504. 10.1137/0153069

[B6] Bartley B. A. Kim K. Medley J. K. Sauro H. M. (2017). Synthetic biology: engineering living systems from biophysical principles. *Biophys. J.* 112 1050–1058. 10.1016/j.bpj.2017.02.013 28355534 PMC5376109

[B7] Baym M. Stone L. K. Kishony R. (2016). Multidrug evolutionary strategies to reverse antibiotic resistance. *Science* 351:aad3292. 10.1126/science.aad3292 26722002 PMC5496981

[B8] Beach R. R. Ricci-Tam C. Brennan C. M. Moomau C. A. Hsu P.-H. Hua B. (2017). Aneuploidy causes non-genetic individuality. *Cell* 169 229–242. 10.1016/j.cell.2017.03.021 28388408 PMC5441241

[B9] Ben-Ami R. Zimmerman O. Finn T. Amit S. Novikov A. Wertheimer N. (2016). Heteroresistance to Fluconazole is a continuously distributed phenotype among Candida glabrata clinical strains associated with in vivo persistence. *mBio* 7 e616–e655. 10.1128/mBio.00655-16 27486188 PMC4981708

[B10] Berger S. (2007). The complex language of chromatin regulation during transcription. *Nature* 447 407–412. 10.1038/nature05915 17522673

[B11] Berman J. Krysan D. J. (2020). Drug resistance and tolerance in fungi. *Nat. Rev. Microbiol.* 18 319–331. 10.1038/s41579-019-0322-2 32047294 PMC7231573

[B12] Blake W. J. Balázsi G. Kohanski M. A. Isaacs F. J. Murphy K. F. Kuang Y. (2006). Phenotypic consequences of promoter-mediated transcriptional noise. *Mol. Cell.* 24 853–856. 10.1016/j.molcel.2006.11.003 17189188

[B13] Bódi Z. Farkas Z. Nevozhay D. Kalapis D. Lázár V. Csörgö B. (2017). Phenotypic heterogeneity promotes adaptive evolution. *PLoS Biol.* 15:e2000644. 10.1371/journal.pbio.1002607 28486496 PMC5423553

[B14] Brauner A. Fridman O. Gefen O. Balaban N. Q. (2016). Distinguishing between resistance, tolerance and persistence to antibiotic treatment. *Nature* 14 320–330. 10.1038/nrmicro.2016.34 27080241

[B15] Brock A. Chang H. Huang S. (2009). Non-genetic heterogeneity-a mutation-independent driving force for the somatic evolution of tumors. *Nat. Rev. Genet.* 10 336–342. 10.1038/nrg2556 19337290

[B16] Camellato B. (2018). *Gene regulatory networks are a mechanism for drug resistance.* Master’s thesis, (Canada: University of Ottawa).

[B17] Camellato B. Roney I. J. Azizi A. Charlebois D. Kærn M. (2019). Engineered gene networks enable non-genetic drug resistance and enhances cellular robustness. *Eng. Biol.* 3 72–79. 10.1049/enb.2019.0009

[B18] Cameron D. E. Bashor C. J. Collins J. J. (2014). A brief history of synthetic biology. *Nat. Rev. Microbiol.* 12 381–390. 10.1038/nrmicro3239 24686414

[B19] Charlebois D. A. (2015). Effect and evolution of gene expression noise on the fitness landscape. *Phys. Rev. E.* 92:022713. 10.1103/PhysRevE.92.022713 26382438

[B20] Charlebois D. A. Abdennnur N. Kærn M. (2011). Gene expression noise facilitates adaptation and drug resistance independently of mutation. *Phys. Rev. Lett.* 107:218101. 10.1103/PhysRevLett.107.218101 22181928

[B21] Charlebois D. A. Balázsi G. (2019). Modeling cell population dynamics. *In Silico Biol.* 13 21–39. 10.3233/ISB-180470 30562900 PMC6598210

[B22] Charlebois D. A. Balázsi G. Kærn M. (2014). Coherent feedforward transcriptional regulatory motifs enhance drug resistance. *Phys. Rev. E.* 89:052708. 10.1103/PhysRevE.89.052708 25353830 PMC5749921

[B23] Dar R. D. Hosmane N. N. Arkin M. R. Siliciano R. F. Weinberger L. S. (2014). Screening for noise in gene expression identifies drug synergies. *Science* 344 1392–1396. 10.1126/science.1250220 24903562 PMC4122234

[B24] de Kraker M. E. A. Stewardson A. J. Harbarth S. (2016). Will 10 Million People Die a Year due to Antimicrobial Resistance by 2050? *PLoS Med.* 13:e1002184. 10.1371/journal.pmed.1002184 27898664 PMC5127510

[B25] Diao J. Charlebois D. A. Nevozhay D. Bodi Z. Csaba P. Balázsi G. (2016). Efflux pump control alters synthetic gene circuit function. *ACS Synth. Biol.* 5 619–631. 10.1021/acssynbio.5b00154 27111147 PMC5752098

[B26] El Karoui M. Hoyos-Flight M. Fletcher L. (2019). Future trends in synthetic biology-a report. *Front. Bioeng. Biotechnol.* 7:175. 10.3389/fbioe.2019.00175 31448268 PMC6692427

[B27] Farquhar K. S. Charlebois D. A. Szenk M. Cohen J. Nevozhay D. Balázsi G. (2019). Role of network-mediated stochasticity in mammalian drug resistance. *Nat. Commun.* 10:2766. 10.1038/s41467-019-10330-w 31235692 PMC6591227

[B28] Farquhar K. S. Flohr H. Charlebois D. A. (2020). Advancing Drug Resistance Research Through Quantitative Modeling and Synthetic Biology. *arXiv*10.3389/fbioe.2020.583415 PMC7530828 33072732

[B29] Ferrari S. Ischer F. Calabrese D. Posteraro B. Sanguinetti M. Fadda G. (2009). Gain of function mutations in CgPHD1 of Candida glabrata not only mediate antifungal resistance but also enhance virulence. *PLoS Pathog.* 5:e1000268. 10.1371/journal.ppat.1000268 19148266 PMC2607542

[B30] Fraser D. Kærn M. (2009). A chance at survival: gene expression noise and phenotypic diversification strategies. *Mol. Microbiol.* 71 1333–1340. 10.1111/j.1365-2958.2009.06605.x 19220745

[B31] Geiler-Samerotte K. A. Bauer C. R. Li S. Ziv N. Gresham D. Siegal M. L. (2013). The details in the distributions: why and how to study phenotypic variability. *Curr. Opin. Biotechnol.* 24 752–759. 10.1016/j.copbio.2013.03.010 23566377 PMC3732567

[B32] Gibson D. G. Young L. Chuang R.-Y. Venter J. C. Hutchison C. A. III. Smith H. O. (2009). Enzymatic assembly of DNA molecules up to several hundred kilobases. *Nat. Methods* 6 343–345. 10.1038/NMETH.1318 19363495

[B33] González C. Ray J. C. J. Manhart M. Adams R. M. Nevozhay D. Morozov A. V. (2015). Stress-response balance drives the evolution of a network module and its host genome. *Mol. Syst. Biol.* 11:827. 10.15252/msb.20156185 26324468 PMC4562500

[B34] Gottesman M. M. Fojo T. Bates S. E. (2002). Multidrug resistance in cancer: role of ATP-dependent transporters. *Nat. Rev. Cancer* 2 48–58. 10.1038/nrc706 11902585

[B35] Gouda M. K. Manhart M. Balázsi G. (2019). Evolutionary regain of lost gene circuit function. *Proc. Natl. Acad. Sci. USA* 116 25162–25171. 10.1073/pnas.1912257116 31754027 PMC6911209

[B36] Henson M. A. (2003). Dynamic modeling of microbial cell populations. *Curr. Opin. Biotech.* 14 460–467. 10.1016/S0958-1669(03)00104-6 14580574

[B37] Inde Z. Dixon S. J. (2018). The impact of non-genetic heterogeneity on cancer cell death. *Crit. Rev. Biochem. Mol. Biol.* 53 99–114. 10.1080/10409238.2017.1412395 29250983 PMC6089072

[B38] Jusiak B. Cleto S. Perez-Piñera P. Lu T. K. (2016). Engineering synthetic gene circuits in living cells with CRISPR technology. *Trends Biotechnol.* 34 353–547. 10.1016/j.tibtech.2015.12.014 26809780

[B39] Kolaczkowski M. Kolaczoska A. Luczynski J. Witek S. Goffeau A. (1998). In Vivo characterization of the drug resistance profile of the major ABC transporters and other components of the yeast pleiotropic drug resistance network. *Microb. Drug. Resist.* 4 143–158. 10.1089/mdr.1998.4.143 9818966

[B40] Kussell E. Kishony R. Balaban N. Q. Leibler S. (2005). Bacterial Persistence: A Model of Survival in Changing Environments. *Genetics* 169 1807–1814. 10.1534/genetics.104.035352 15687275 PMC1449587

[B41] Langevin A. M. Dunlop M. J. (2018). Stress introduction rate alters the benefit of AcrAB-TolC efflux pumps. *J. Bacteriol.* 200 e517–e525. 10.1128/JB.00525-17 29038251 PMC5717160

[B42] Lavi O. Gottesman M. M. Levy D. (2012). The dynamics of drug resistance: A mathematical perspective. *Drug Resist. Updat.* 15 90–97. 10.1016/j.drup.2012.01.003 22387162 PMC3348255

[B43] Levin-Reisman I. Ronin I. Gefen O. Braniss I. Shoresh N. Balaban N. Q. (2017). Antibiotic tolerance facilitates the evolution of resistance. *Science* 355 826–830. 10.1126/science.aaj2191 28183996

[B44] Misra S. Ghatak S. Toole B. P. (2005). Regulation of MDR1 Expression and Drug Resistance by a Positive Feedback Loop Involving Hyaluronan, Phosphoinositide 3-Kinase, and ErbB2. *J. Biol. Chem.* 280 20310–20315. 10.1074/jbc.M500737200 15784621

[B45] Mitchell S. Roy K. Zangle T. A. Hoffmann A. (2018). Nongenetic origins of cell-to-cell variability in B lymphocyte proliferation. *Proc. Natl. Acad. Sci. USA* 115 E2888–E2897. 10.1073/pnas.1715639115 29514960 PMC5866559

[B46] Mondon P. Petter R. Amalfitano G. Luzzati R. Concia E. Polacheck I. (1999). Heteroresistance to fluconazole and voriconazole in Cryptococcus neoformans. *Antimicrob. Agents Chemother.* 43 1856–1861. 10.1128/AAC.43.8.1856 10428902 PMC89380

[B47] Nevozhay D. Adams R. M. Itallie E. V. Bennett M. R. Balázsi G. (2012). Mapping the environmental fitness landscape of a synthetic gene circuit. *PLoS Comput. Biol.* 8:1–17. 10.1371/journal.pcbi.1002480 22511863 PMC3325171

[B48] Nevozhay D. Zal T. Balázsi G. (2013). Transferring a synthetic gene circuit from yeast to mammalian cells. *Nat. Commun.* 4 1–11. 10.1038/ncomms2471 23385595 PMC3573884

[B49] Nissim L. Perli S. D. Fridkin A. Perez-Pinera P. Lu T. K. (2014). Multiplexed and programmable regulation of gene networks with an integrated RNA and CRISPR/Cas toolkit in human cells. *Mol. Cell.* 54 698–710. 10.1016/j.molcel.2014.04.022 24837679 PMC4077618

[B50] Ochman H. Lawrence J. G. Groisman E. A. (2000). Lateral gene transfer and the nature of bacterial innovation. *Nature* 405 299–304. 10.1038/35012500 10830951

[B51] O’Neill J. The Review on Antimicrobial Resistance. (2016). *Tackling Drug-Resistant Infections Globally: Final Report and Recommendations.* London: Wellcome Trust.

[B52] Rosenberg A. Ene I. V. Bibi M. Zakin S. Segal E. S. Ziv N. (2018). Antifungal tolerance is a subpopulation effect distinct from resistance and is associated with persistent candidemia. *Nat. Commun.* 9:2470. 10.1038/s41467-018-04926-x 29941885 PMC6018213

[B53] Sakurai Y. Hori Y. (2018). Optimization-based synthesis of stochastic biocircuits with statistical specifications. *J. R. Soc. Interface* 15:20170709. 10.1098/rsif.2017.0709 29321266 PMC5805972

[B54] Salgia R. Kulkarni P. (2018). The genetic/non-genetic duality of drug ‘resistance’ in cancer. *Trends Cancer* 4 110–118. 10.1016/j.trecan.2018.01.001 29458961 PMC5822736

[B55] Santos-Moreno J. Schaerli Y. (2019). A framework for the modular and combinatorial assembly of synthetic gene circuits. *ACS Synth. Biol.* 8 1691–1697. 10.1021/acssynbio.9b00174 31185158

[B56] Sears D. Schwartz B. S. (2017). Candida Auris: an emerging multidrug-resistant pathogen. *Int. J. Infect. Dis.* 63 95–98. 10.1016/j.ijid.2017.08.017 28888662

[B57] Shaffer S. M. Dunagin M. C. Torborg S. R. Torre E. A. Emert B. Krepler C. (2017). Rare cell variability and drug-induced reprogramming as a mode of cancer drug resistance. *Nature* 546 431–435. 10.1038/nature22794 28607484 PMC5542814

[B58] Tripathi S. Chakraborty P. Levine H. Jolly M. K. (2020). A mechanism for epithelial-mesenchymal heterogeneity in a population of cancer cells. *PLoS Comput. Biol.* 16:e1007619. 10.1371/journal.pcbi/1007619 32040502 PMC7034928

[B59] van Boxtel C. van Heerden J. H. Nordholt N. Schmidt P. Bruggeman F. J. (2017). Taking chances and making mistakes: non-genetic phenotypic heterogeneity and its consequences for surviving in dynamic environments. *J. R. Soc. Interface* 14:20170141. 10.1098/rsif.2017.0141 28701503 PMC5550968

[B60] Vasan N. Baselga J. Hyman D. M. (2019). A view on drug resistance in cancer. *Nature* 575 299–309. 10.1038/s41586-019-1730-1 31723286 PMC8008476

[B61] Wang T. Kunze C. Dunlop M. J. (2019). Salicylate increases fitness cost associated with MarA-mediated antibiotic resistance. *Biophys. J.* 117, 563–571. 10.1016/j.bpj.2019.07.005 31349991 PMC6697527

[B62] Wirth D. Hauser H. (2004). Flp-mediated integration of expression cassettes into FRT-tagged chromosomal loci in mammalian cells. *Methods Mol. Biol.* 267 467–476. 10.1385/1-59259-774-2:467 15269443

[B63] World Health Organization. (2014). *Antimicrobial Resistance: Global report of surveillance.* Switzerland: WHO.

[B64] Wuyts J. Van Dijck P. Holtappels M. (2018). Fungal persister cells: the basis for recalcitrant infections? *PLoS Pathog.* 14:e1007301. 10.1371/journal.ppat.1007301 30335865 PMC6193731

[B65] Zhang Y. Yew W. W. Barer M. R. (2012). Targeting persisters for tuberculosis control. *Antimicrob. Agents Chemother.* 56 2223–2230. 10.1128/AAC.062886211 22391538 PMC3346619

[B66] Zhuravel D. Fraser D. St-Pierre S. Tepliakova L. Pang W. L. Hasty J. (2010). Phenotypic impact of regulatory noise in cellular stress-response pathways. *Syst. Synth. Biol.* 4 105–116. 10.1007/s11693-010-9055-2 20805931 PMC2923296

